# Letter to the Editor: “Exercise Training Adaptations in Metabolic Syndrome Individuals on Chronic Statin Treatment”

**DOI:** 10.1210/clinem/dgaa416

**Published:** 2020-06-29

**Authors:** Neeltje A E Allard, Maria T E Hopman, Silvie Timmers

**Affiliations:** 1 Radboud Institute for Health Sciences, Department of Physiology, Radboud University Medical Center, Nijmegen, The Netherlands; 2 Human and Animal Physiology, Wageningen University, Wageningen, The Netherlands

With great interest we read the article by Morales-Palomo et al (1), in which the therapeutic effects of a 16-week high-intensity interval training (HIIT) were studied in chronic statin users compared with nonstatin using controls, all diagnosed with metabolic syndrome. The HIIT program resulted in similar improvements in fat metabolism and mitochondrial enzyme activities in both groups, but improvement in cardiorespiratory fitness was attenuated in the statin users. Studying these training adaptations in chronic statin users is very informative because previous training studies have only been performed in statin-naïve (2) or statin-tolerating subjects (3). However, we question whether HIIT is the most suitable exercise program for statin users. In addition, it is unknown whether the statin users were experiencing muscle symptoms before exercise and if these were aggravated by the training program.

Cardiovascular risk reduction is best achieved with the combination of both physical activity and statin treatment (4). Even though statins are well tolerated, statin-associated muscle symptoms (SAMS) are the most reported adverse effect. SAMS are an important cause for nonadherence to statin treatment (5). Physical activity may exacerbate SAMS, making a physically active lifestyle difficult for statin users. High-intensity training and acute eccentric and strenuous exercise are especially prone to worsen muscle symptoms (6). In contrast, physical activity at moderate intensity may counteract statin-induced myocellular changes by improving mitochondrial function and attenuate SAMS (7). Because no information is supplied on muscle-related problems experienced during the exercise program, this study does not provide insight whether HIIT was tolerated well by the statin users.

In addition, the statin users were not characterized by the presence or absence of SAMS before exercise training. We recently showed that disturbances in mitochondrial function are more pronounced in symptomatic statin users compared with asymptomatic statin users. Furthermore, we observed an inverse correlation between pain intensity in symptomatic statin users and mitochondrial complex III activity (8). This provides evidence that symptomatic and asymptomatic statin users are affected differently by statins, which raises the possibility that exercise adaptations could be different between these groups.

We believe that it is important to record and minimize SAMS in patients at risk for cardiovascular diseases, to prevent nonadherence to statins while maintaining a physically active lifestyle. A tailored exercise program for statin users should take the latter information into account and monitor that muscle complaints are not worsened because of the exercise program. We and others (7) feel this may be best achieved with exercises of moderate intensity, although it is necessary to explore whether moderate intensity training leads to similar training adaptations as HIIT. Moreover, we want to highlight the importance of distinguishing asymptomatic from symptomatic statin users before exercise interventions because differences in energy metabolism are observed. We propose that further research should investigate adaptations to moderate intensity training in chronic symptomatic and asymptomatic statin users while monitoring muscle symptoms.

We want to thank the authors for their important contribution and hope that they are able to address our considerations.

## Additional Information

***Disclosures:*** The authors have no relationships or conflicts to disclose.
